# Combined tibial plateau leveling osteotomy and autologous conditioned serum for functional recovery and delayed osteoarthritis progression in dogs with cranial cruciate ligament rupture

**DOI:** 10.3389/fvets.2026.1902443

**Published:** 2026-09-10

**Authors:** Caihui Ji, Guorong Gao, Ganzhen Deng

**Affiliations:** 1 College of Veterinary Medicine, Huazhong Agricultural University, Wuhan, Hubei, China; 2 Department of Surgery, Ruipeng Pet Hospital, Shenzhen, Guangdong, China

**Keywords:** autologous conditioned serum, canine cranial cruciate ligament rupture, inflammatory cytokines, osteoarthritis, tibial plateau leveling osteotomy

## Abstract

**Background:**

Cranial cruciate ligament rupture (CCLR) is the leading cause of stifle joint instability, chronic pain, and osteoarthritis (OA) in dogs. Although tibial plateau leveling osteotomy (TPLO) is the standard surgical treatment, postoperative inflammation and secondary OA progression remain major challenges. Autologous conditioned serum (ACS) is a biological therapy with anti-inflammatory and chondroprotective properties.

**Methods:**

In this prospective, randomized controlled trial, 60 stifles (48 client-owned dogs) with complete CCLR were allocated equally to three groups (*n* = 20 each): ACS-only, TPLO-only, and TPLO+ACS. Assessments at preoperative baseline (D0) and postoperative days 30–180 included orthopedic assessment scores (OAS), radiographic OA grading, and synovial fluid cytokine levels (IL-1β, IL-10, TNF-α). A subset of 15 stifles was followed up to 720 days for long-term OA progression.

**Results:**

All groups showed significant functional improvement (*p* < 0.001). The TPLO+ACS group achieved the greatest OAS reduction (ΔOAS = 7.29 points) and the lowest D180 score (0.57 ± 0.16), outperforming both monotherapy groups (*p* < 0.05). Short-term OA progression ( ≤ 180 days) did not differ significantly; however, at 720 days, TPLO+ACS maintained significantly lower OA scores (1.8 ± 0.4) than ACS (2.3 ± 0.5) and TPLO (2.2 ± 0.7) groups (*p* < 0.05). Synovial inflammatory cytokine analysis revealed that TPLO+ACS most effectively suppressed pro-inflammatory cytokines (IL-1β, TNF-α) and sustained anti-inflammatory IL-10 levels during the early-to-mid postoperative phase.

**Conclusion:**

TPLO combined with ACS provides great improvement for canine CCLR by restoring biomechanical stability and modulating the intra-articular inflammatory microenvironment. This integrated approach enhances functional recovery and retards OA progression.

## 1 Introduction

Cranial cruciate ligament rupture (CCLR) is the most common orthopedic disorder afflicting the canine stifle joint, and causes lameness, pain, joint instability, and progressive osteoarthritis (OA). The underlying pathogenesis is frequently rooted in progressive degenerative joint disease and ligament failure, which predisposes the tissue to mechanical failure (1, 2). In this milieu, resident fibroblasts ignite the inflammatory cascade, orchestrating the recruitment of peripheral inflammatory cells—the principal reservoirs of pro-inflammatory cytokines such as IL-1β and TNF-α (1, 2). These mediators fuel a vicious cycle of collagen degradation, synovial inflammation, and cartilage erosion. Counterbalancing this assault, anti-inflammatory mediators (e.g., IL-1 receptor antagonist, IL-1Ra) serve to quell the cascade and limit tissue destruction. Given the heightened susceptibility of giant and large breeds, early surgical intervention is strongly advocated to restore limb function and avert irreversible cartilage damage (2).

Tibial plateau leveling osteotomy (TPLO) remains the gold-standard surgical procedure for CCLR in dogs, effectively neutralizing cranial tibial thrust and conferring long-term joint stability. However, clinical outcomes are frequently compromised by postoperative synovitis, persistent pain, and the insidious progression of OA (3, 4). This has fueled a growing interest in biotherapeutic adjuvants capable of modulating inflammation and safeguarding chondral structures. Autologous conditioned serum (ACS), replete with anti-inflammatory cytokines (e.g., IL-1Ra) and growth factors, represents a promising candidate to suppress inflammation, alleviate pain, and potentially restore cartilage integrity. While ACS has demonstrated efficacy in ameliorating canine OA and joint injuries (5), robust clinical evidence validating its synergistic utility with TPLO is lacking. Accordingly, this study endeavors to rigorously evaluate the clinical efficacy of the TPLO+ACS combination. We hypothesize that this integrated approach will yield superior outcomes in early functional recovery, postoperative analgesia, and the mitigation of OA progression compared to monotherapy.

## 2 Materials and methods

### 2.1 Subjects and random allocation

This prospective, randomized controlled trial enrolled 60 stifles (48 client-owned dogs) presented with unilateral or bilateral CCLR. All subjects were recruited from Ruipeng Pet Hospital. Inclusion criteria required a definitive diagnosis of complete CCLR confirmed by orthopedic examination (e.g., cranial drawer test) and radiography, while excluding patients with systemic diseases, infections, or other stifle pathologies. Following randomization, subjects were allocated to one of three groups (*n* = 20 per group): ACS group received intra-articular ACS monotherapy; TPLO group underwent TPLO alone; and Group TPLO+ACS group was treated with the combined TPLO and ACS protocol.

### 2.2 Surgical and biologic treatment protocols

All operative interventions were completed by a single board-certified veterinary surgeon under standardized general anesthesia combined with multimodal perioperative analgesia. A conventional medial access route to the proximal tibia was employed, spanning from the mid-diaphysis to the tibial plateau without arthrotomy. TPLO was performed to reach a post-surgical tibial plateau angle (TPA) of 6°, with osteotomy stabilization attained via a locking bone plate and screws.

In parallel, the preparation of ACS was conducted under under aseptic conditions. Fifteen milliliters of venous blood were collected in specialized Orthokine^®^ Vet Irap collection tubes (Orthogen Veterinary GmbH, DUS, DE) and subjected to incubation at 37 °C for 4–6 h to promote the release of anti-inflammatory cytokines. Subsequent performance of centrifugation at 1500 × g for 3 min led to the acquisition of the serum. At the end of surgery, 2.5 mL of the processed ACS was injected into the articular cavity of dogs in the ACS and TPLO+ACS groups. In contrast, experimental subjects in the TPLO group were given an equal volume of aseptic 0.9% sodium chloride solution.

### 2.3 Postoperative management and assessment

Postoperative care followed a standardized protocol for all enrolled groups, including 4 weeks of activity restriction and a 2-week regimen of multimodal analgesia. Serial evaluations were performed at preoperative baseline (D0) and Days 30, 60, 90, 120, 150, and 180 postoperatively, respectively. These evaluations included OAS, radiographic OA scores, and synovial cytokine levels. OAS included lameness classification, pain scoring, measurement of stifle joint range of motion (ROM); (Table 1), and radiographic OA grading based on established standards (6, 7) (Table 2), radiographic OA scores were assessed by a board-certified veterinary radiologist blinded to treatment group. All physical examinations, including general health and orthopedic assessments, were performed by a single veterinarian at each follow-up interval. Gait analysis was performed using video recordings of dogs during free walking. Lameness was quantified using a modified OAS (8), with a maximum possible score of 11 points (Table 1). The mean OAS change (ΔOAS) was calculated from preoperative baseline (D0) measurements to each subsequent follow-up time point. Stifle joint ROM was measured using a goniometer, with reference to the established normal value of 121° (9). To ensure accuracy, the mean of three consecutive measurements was recorded for statistical analysis.

**Table 1:** Scoring system for the orthopedic assessment score (OAS).

Clinical parameter	Scoring criteria	Score
Lameness	Mild, intermittent lameness	1
	Obvious weight-bearing lameness	2
	Severe weight-bearing lameness	3
	Intermittent non-weight-bearing lameness	4
	Persistent non-weight-bearing lameness	5
Range of motion	ROM reduced by 10%−20%	1
	ROM reduced by 20%−50%	2
	ROM reduced by >50%	3
Pain	Mild pain (dog turns head in response)	1
	Moderate pain (dog withdraws limb or attempts to move away)	2
	Severe pain (dog vocalizes and exhibits aggression)	3

**Table 2:** Kellgren-lawrence scoring system for osteoarthritis.

Score	Grade	Description
0	Normal	No radiographic features of osteoarthritis
1	mild	Questionable joint space narrowing, possible osteophyte formation
2	Moderate	Definite osteophyte formation, possible joint space narrowing
3	severe	Definite joint space narrowing, moderate osteophyte formation, mild subchondral sclerosis
4	Very severe	Severe joint space narrowing, extensive osteophyte formation, and marked subchondral sclerosis

For synovial cytokine analysis, synovial fluid samples were aspirated from the affected stifle joints to quantify concentrations of key inflammatory cytokines (IL-1β, IL-10, and TNF-α) using commercial ELISA kits (Catalog #MM-1492O2, MM-1550O2, MM-36988O2; Jiangsu Enzyme Immunoassay Biotech Co., Ltd., China).

### 2.4 Statistical analysis

The SPSS 26.0 software was used to analyze the data statistically. Differences between groups were compared by the use of an independent samples *t*-test, chi-square test, and repeated-measures ANOVA. A *p*-value < 0.05 was considered statistically significant.

## 3 Results

### 3.1 Population characteristics of the study cohort

60 stifles (48 client-owned dogs) with CCLR were enrolled in this prospective, randomized clinical study. The cohort comprised a diverse range of breeds, including Poodles (11/48, 22.9%), Bichon Frises (8/48, 16.7%), Corgis (10/48, 20.8%), Golden Retrievers (5/48, 10.4%), Labrador Retrievers (6/48, 12.5%), Alaskan Malamutes (3/48, 6.2%), and Border Collies (5/48, 10.4%). These dogs varied in age, body weight, and disease duration. Of the 60 stifles evaluated, 36 (60%) were unilateral and 24 (40%) were bilateral. Among the 48 dogs, 29 (60.4%) were male and 19 (39.6%) were female. The means of age, body weight, and TPA for each group are summarized in Table 3.

**Table 3:** The mean age, body weight, and tibial plateau angle (TPA) of the three groups.

Group	Stifles (*n*)	Mean age (years)	Mean Body Weight (kg)	Mean TPA (°)	
				Preoperative	Postoperative
ACS	20	6.3 ± 2.3	11.3 ± 6.5^a^	27.6 ± 2.1	27.6 ± 2.1
TPLO	20	5.3 ± 1.9	17.9 ± 11.5^ab^	29.6 ± 1.9	6.1 ± 1.6
TPLO+ACS	20	5.9 ± 2.1	21.6 ± 8.7^b^	30.1 ± 2.6	6.3 ± 1.3

Within each column, values with different superscript letters differ significantly (*p* < 0.05). Postoperative TPA remained unchanged in the ACS group due to the non-osteotomy nature of the intervention.

Although the mean ages of the three treatment groups were comparable (5.3–6.3 years), significant intergroup differences (*p* < 0.05) in mean body weight (11.3–21.6 kg) were noted; therefore, body weight was included as a covariate in the outcome analyses. Specifically, the TPLO+ACS group (21.6 ± 8.7 kg) was significantly heavier than the ACS group (11.3 ± 6.5 kg, *p* < 0.05), and the TPLO group (17.9 ± 11.5 kg) also weighed significantly more than the ACS group (*p* < 0.05). However, no significant difference was observed between the TPLO and TPLO+ACS groups (*p* > 0.05). The presence of all groups with pathological preoperative tibial plateau angles (>25°) and the uniformity of postoperative correction to about 6° (mean correction >23°) in both surgical groups ensured accurate and stable osteotomy.

### 3.2 Clinical outcomes

**Figure 1:**
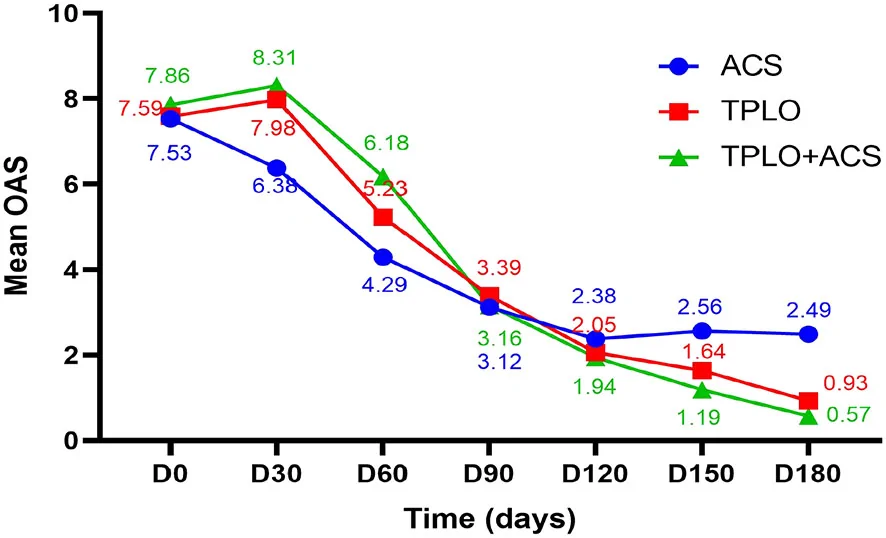
Changes in the mean orthopedic assessment scores of the three groups. The results are presented as the mean OAS score, lower scores correspond to a better orthopedic function, OAS, orthopedic assessment score, ACS, autologous conditioned serum, TPLO, tibial plateau leveling osteotomy, *n* = 20 per group.

There were 60 stifles randomly allocated in the ACS, TPLO, or TPLO+ACS groups. The OAS system was used to measure orthopedic function at the preoperative period (D0) and days 30, 60, 90, 120, 150, and 180 after surgery, with a mean OAS value plotted by time (Figure 1).

Repeated measures ANOVA revealed a significant main effect of time on orthopedic function across all groups (*p* < 0.001). From D0 to D180, the TPLO+ACS group showed the greatest improvement in OAS (ΔOAS = 7.29 points), which was significantly greater than that in the TPLO (ΔOAS = 6.46 points) and ACS (ΔOAS = 5.04 points) groups. Significant intergroup differences were observed at specific time points: at D30, the mean OAS in the ACS group was significantly lower than in the two surgical groups (*p* < 0.05). By D180, the TPLO+ACS group achieved the lowest OAS score (0.57 ± 0.16), followed by TPLO (0.93 ± 0.19) and ACS (2.49 ± 0.21), with all differences reaching statistical significance (*p* < 0.05).

### 3.3 Radiographic OA progression

Based on the radiographic OA scoring system, the mean OA scores of the 60 stifle joints across the three groups were evaluated at prooerative baseline (D0) and days from 30 to 180 postoperatively, and the results were presented in Table 4. No significant changes in the mean osteoarthritis score were observed over the 180-day postoperative period in any of the three groups (*p* > 0.05). Furthermore, the interaction effect between time and treatment group was not significant (*p* > 0.05).

**Table 4:** The mean radiographic OA scores of 60 stifles.

Group	Preoperative	Postoperative					
	D0	D30	D60	D90	D120	D150	D180
ACS	1.1 ± 0.4^a^	1.1 ± 0.2^a^	1.1 ± 0.3^a^	1.1 ± 0.4^a^	1.1 ± 0.4^a^	1.1 ± 0.4^a^	1.2 ± 0.5^a^
TPLO	1.2 ± 0.5^a^	1.2 ± 0.5^a^	1.2 ± 0.5^a^	1.2 ± 0.6^a^	1.2 ± 0.7^a^	1.2 ± 0.7^a^	1.2 ± 0.8^a^
TPLO+ACS	1.2 ± 0.4^a^	1.2 ± 0.4^a^	1.2 ± 0.4^a^	1.2 ± 0.5^a^	1.2 ± 0.5^a^	1.2 ± 0.6^a^	1.2 ± 0.4^a^

Within each column, values with different superscript letters differ significantly (*p* < 0.05). *n* = 20 per group.

**Figure 2:**
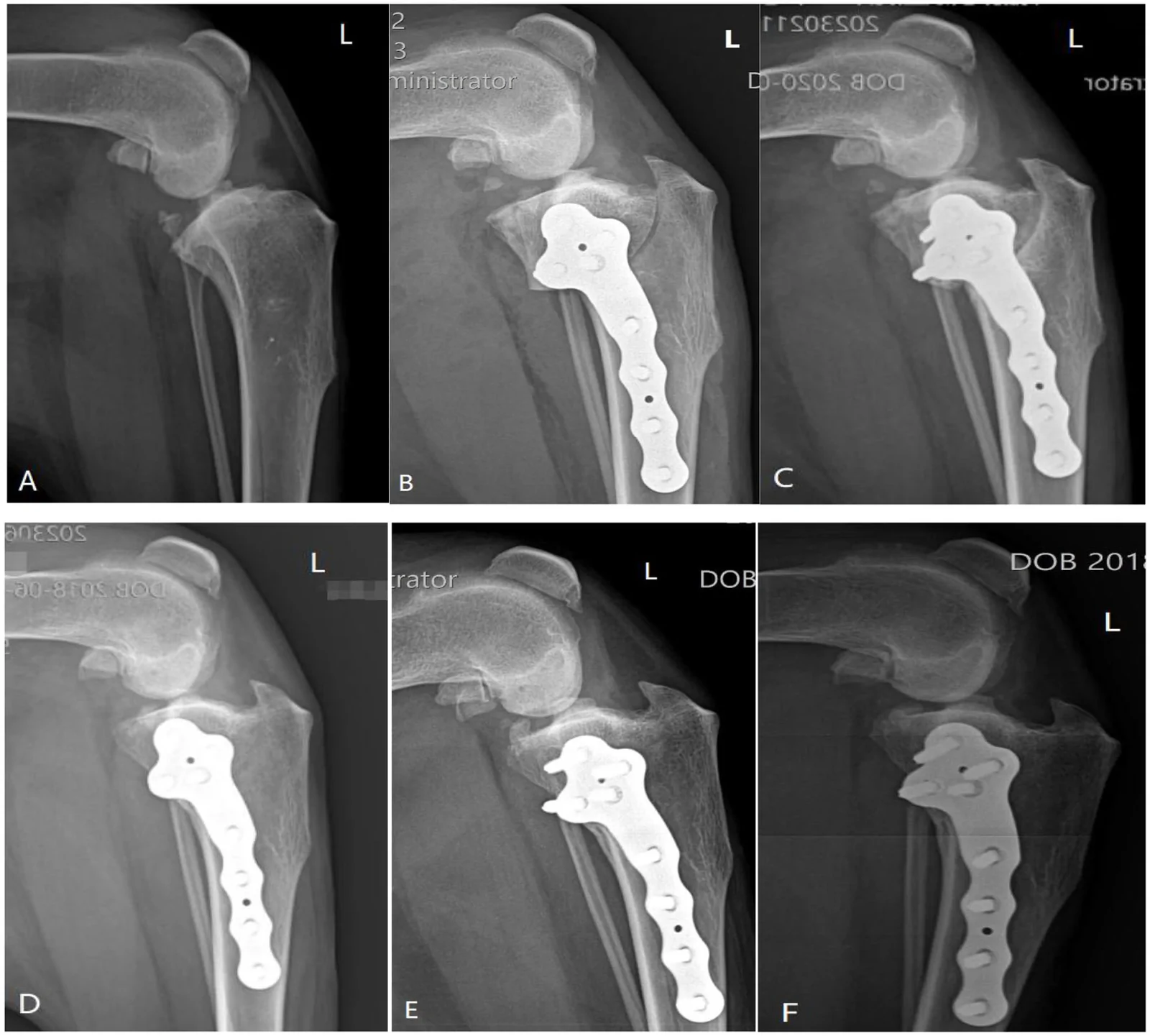
Serial radiographs of left CCLR from TPLO+ACS group. Left stifle radiographs **(A–F)** from a 5-year-old, 42-kg male Alaskan Malamute treated with TPLO+ACS for left CCLR, shown from preoperative to 720 days postoperatively (days 1, 30, 180, 240, and 720) in the mediolateral view. **(A)** Preoperative: marked effusion and cranial tibial subluxation; incipient osteophytes on the patella, femur, and trochlear ridge. **(B)** Immediate postoperative (D1): distinct osteotomy line, plate stabilization, and persistent mild effusion. **(C)** Day 30: moderate effusion; osteotomy line still defined. **(D)** Day 90: blurring of the osteotomy line indicating early healing, with mild effusion. **(E)** Day 180: osteotomy line indistinct, suggesting progressive osseous union, and persistent mild effusion. **(F)** Day 360: complete bony union with obliteration of the osteotomy line; mild osteophyte formation. Overall, radiographic assessment throughout the 2-year follow-up indicated moderate OA with satisfactory bone healing, substantially reduced effusion, no significant osteophyte proliferation, and a stable OA grade of 2.

**Figure 3:**
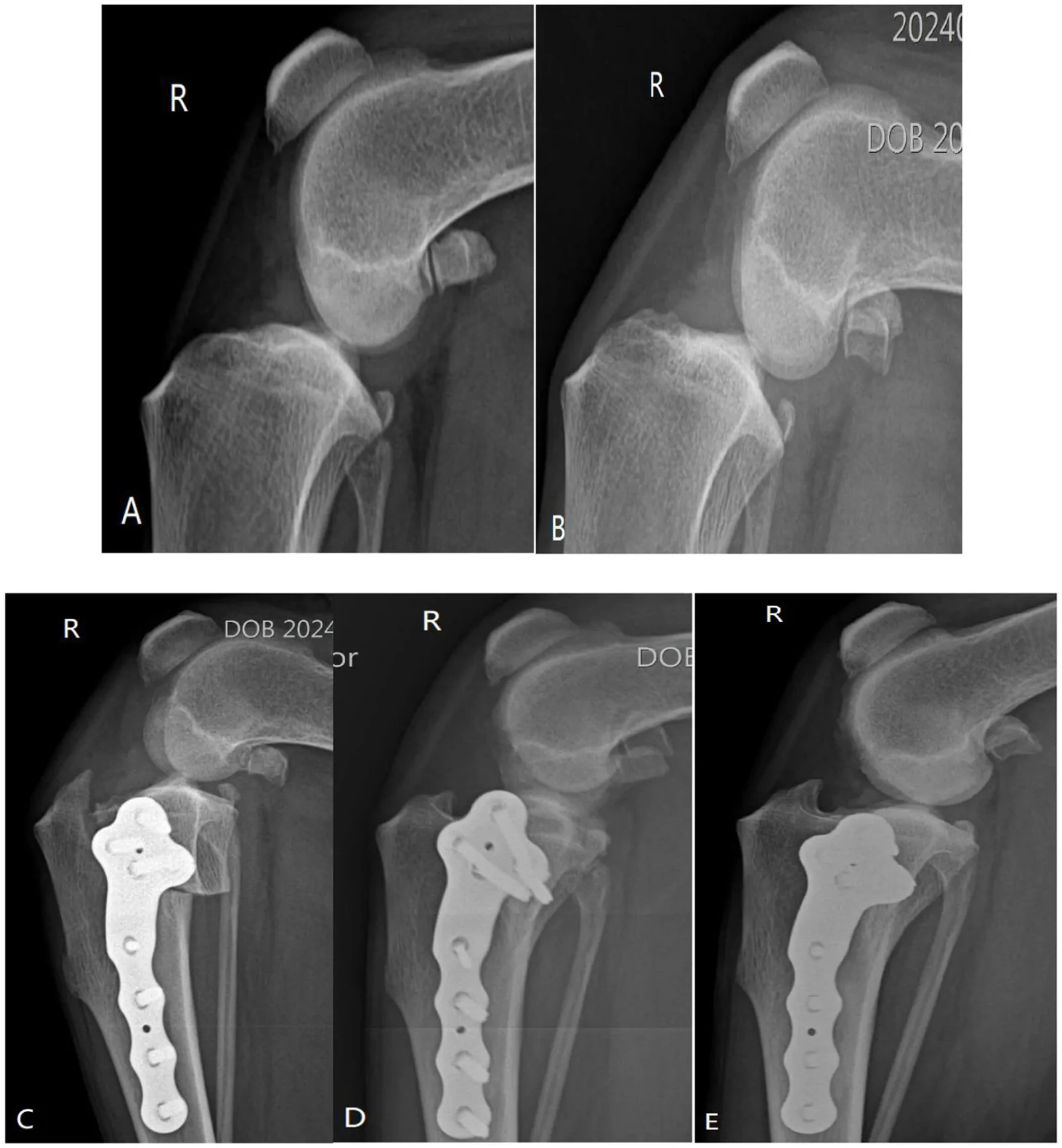
Comparative radiographs of right CCLR from ACS and TPLO+ACS groups. Serial radiographic images **(A–E)** of right CCLR in a 6-year-old, 43-kg male Alaskan Malamute. After receiving ACS monotherapy for 360 days in the right stifle, the dog developed severe hindlimb lameness, prompting a switch to combination TPLO+ACS treatment. Radiographs were obtained in the mediolateral view. **(A)** Before ACS therapy, the radiograph revealed moderate joint effusion, cranial tibial subluxation, and no significant osteophyte formation. **(B)** Radiograph at 360 days post-ACS monotherapy demonstrated severe joint effusion and marked osteophyte formation, which indicated progressive osteoarthritis (OA score: 2). **(C)** Immediate postoperative radiograph from the TPLO+ACS treatment showed a distinct osteotomy line and bone plate fixation, and severe joint effusion was present within the joint cavity. **(D)** Postoperative radiograph at D360 (360 days after TPLO+ACS) revealed complete bony union (obliteration of the osteotomy line), resolution of joint effusion, and no significant progression of osteophytes. **(E)** Radiograph at Day 610 demonstrated no significant joint effusion or further osteophyte progression, which indicated a stable OA grade of 2.

**Figure 4:**
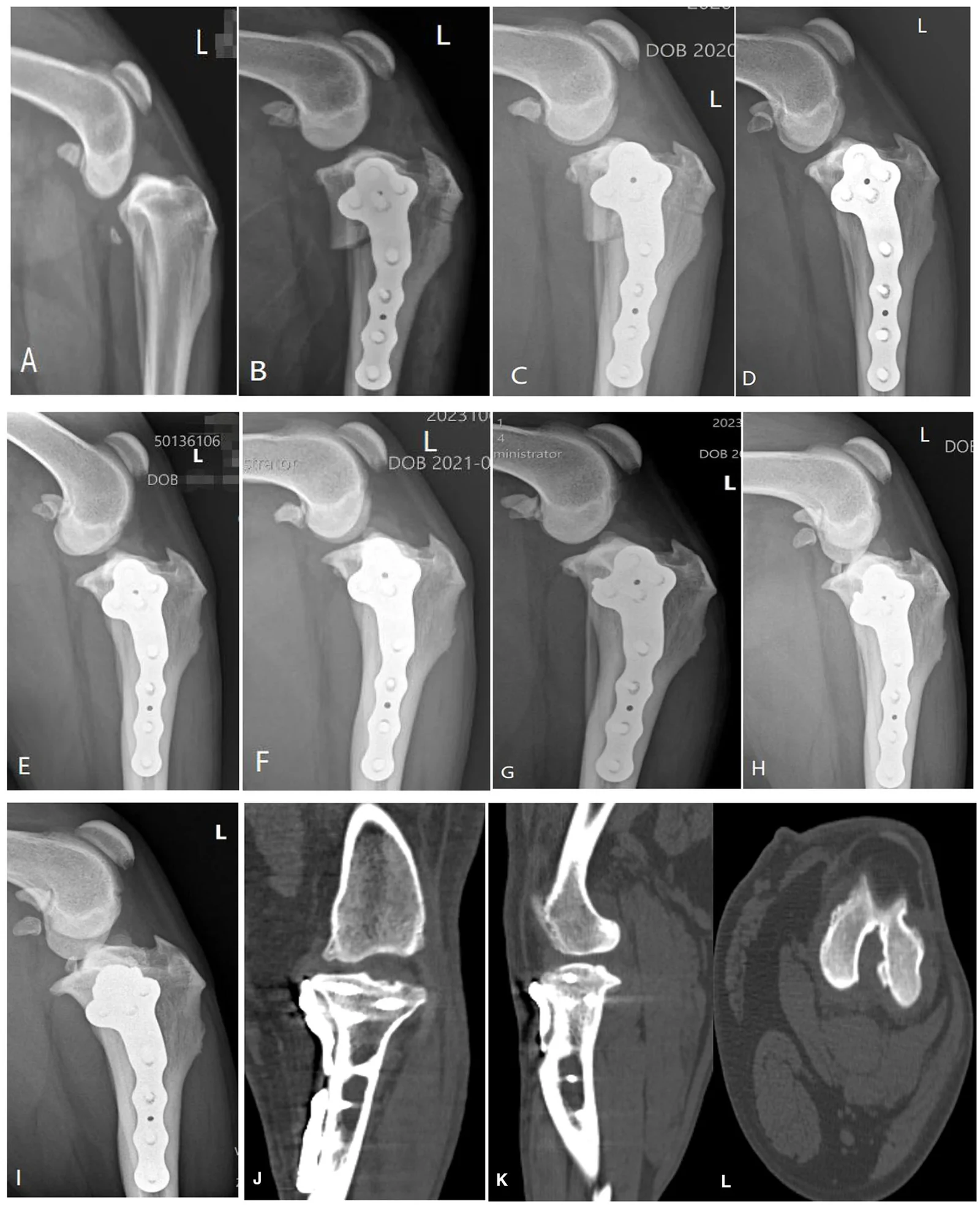
Serial radiographs and CT imaging of left stifle from TPLO group. Serial images **(A–J)** of the left stifle joint illustrated the progression of degenerative changes in a 48-kg, 1-year-old castrated male Alaskan Malamute treated with TPLO for left CCLR. Serial radiographs from the preoperative baseline (D0) to 1,200 days postoperatively demonstrated progression from early arthritic changes to moderate osteoarthritis (OA score: 2). All radiographs were mediolateral views. **(A)** Preoperative radiograph demonstrated cranial tibial subluxation and marked joint effusion, with no evidence of osteophytosis. **(B)** Immediate postoperative radiograph revealed persistent marked joint effusion, a distinct osteotomy line, and plate fixation. **(C)** Radiograph acquired 30 days postoperatively showed severe joint effusion, and the osteotomy line was still clearly defined. **(D)** Radiograph from day 150 showed the osteotomy line indistinct, which indicated osseous union; moderate joint effusion persisted, and no osteophytes were yet apparent. **(E–F)** Radiographs from days 360 and 510, respectively, demonstrated persistent moderate joint effusion; subtle osteophyte formation was first noted at the femoral intercondylar notch on day 510. **(G–H)** Radiographs from days 840 and 960 revealed progressive osteophyte formation that extended. **(I–L)** Radiographs and CT imaging at day 1200 postoperatively showed persistent moderate joint effusion and extensive mature osteophyte development that involved both tibial and femoral condyles. Despite satisfactory bone healing and implant stability, persistent joint effusion and significant osteophyte proliferation were consistently observed.

In contrast, long-term follow-up of the 15 representative stifle joints across the three groups up to 720 days postoperatively showed the mean radiographic OA scores, as summarized in Table 5. Two-way repeated measures ANOVA revealed a significant interaction effect between time and treatment on OA scores (*p* < 0.05). There were no significant differences in OA scores among the three groups at baseline (*p* > 0.05). Within 180 days postoperatively, no significant increases in OA scores were observed in any of the groups (*p* > 0.05). However, starting at 360 days, the OA scores in the ACS group were significantly higher than those in the TPLO+ACS group (*p* < 0.05). By 720 days, the OA score in the TPLO+ACS group (1.8 ± 0.4) was significantly lower than that in the ACS group (2.3 ± 0.5) and the TPLO group (2.2 ± 0.7), with all differences reaching statistical significance (*p* < 0.05). Intragroup comparisons demonstrated that the OA scores at 2 years postoperatively were significantly elevated from baseline in both the ACS and TPLO groups (*p* < 0.05), whereas no significant progression was detected in the TPLO+ACS group (*p* > 0.05). Overall, radiographic osteoarthritis scores were significantly lower in the TPLO+ACS group at 720 days. Representative radiographs from each of the three treatment groups are shown in Figures 2–4.

**Table 5:** The mean radiographic OA scores of 15 stifles.

Group	Stifles (*n*)	Preoperative	Postoperative			
		D0	D180	D360	D450	D720
ACS	5	1.1 ± 0.3^a^	0.9 ± 0.6^a^	1.7 ± 0.4^a^	2.1 ± 0.7^a^	2.3 ± 0.5^a^
TPLO	5	1.1 ± 0.7^a^	1.2 ± 0.9^a^	1.5 ± 0.8^ab^	1.9 ± 0.8^ab^	2.2 ± 0.7^b^
TPLO+ACS	5	1.2 ± 0.5^a^	1.2 ± 0.6^a^	1.3 ± 0.8^b^	1.6 ± 0.7^b^	1.8 ± 0.4^c^

Within each column, values with different superscript letters differ significantly (*p* < 0.05).

### 3.4 Quantification of inflammatory cytokine levels

**Figure 5:**
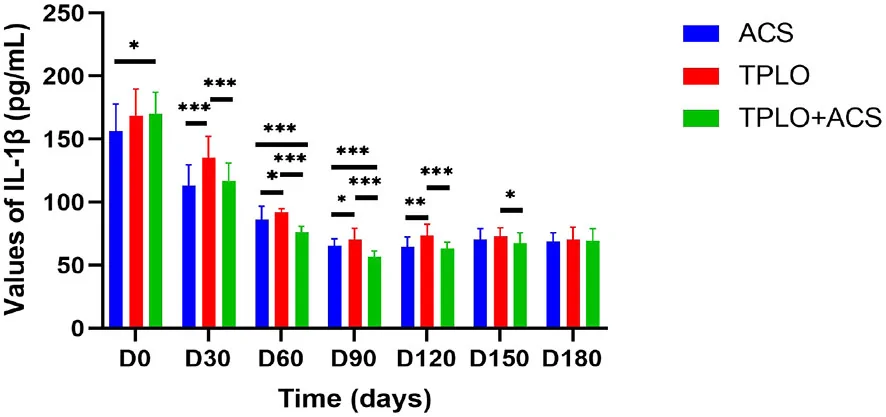
Changes in mean IL-1β in stifle joint synovial fluid. The results are presented as the mean ± SD, and statistical significance was calculated by *t* tests for two groups and one-way ANOVA for three groups. *p < 0.05, **p < 0.01, ***p < 0.001, ACS, autologous conditioned serum, TPLO, tibial plateau leveling osteotomy, *n* = 20 per group.

At baseline (D0), no significant differences in synovial IL-1β concentrations (160–170 pg/mL) were observed among the ACS, TPLO, and TPLO+ACS groups. Over the 180-day postoperative period, IL-1β levels in all groups exhibited a general downward trend. However, the TPLO+ACS group demonstrated significantly lower IL-1β values compared to the TPLO group at D30, D60, D90, D120, and D150 (*p* < 0.001, *p* < 0.01, respectively). Furthermore, the TPLO+ACS group showed significantly reduced IL-1β levels compared to the ACS group at D30, D60, D90, and D120 (*p* < 0.05). Notably, the ACS group also exhibited significantly lower IL-1β concentrations than the TPLO group during the early postoperative phase (D30 and D60, *p* < 0.05). By D180, IL-1β levels in all three groups decreased to a comparable range (70 pg/mL), with no statistically significant differences observed among the groups (Figure 5).

**Figure 6:**
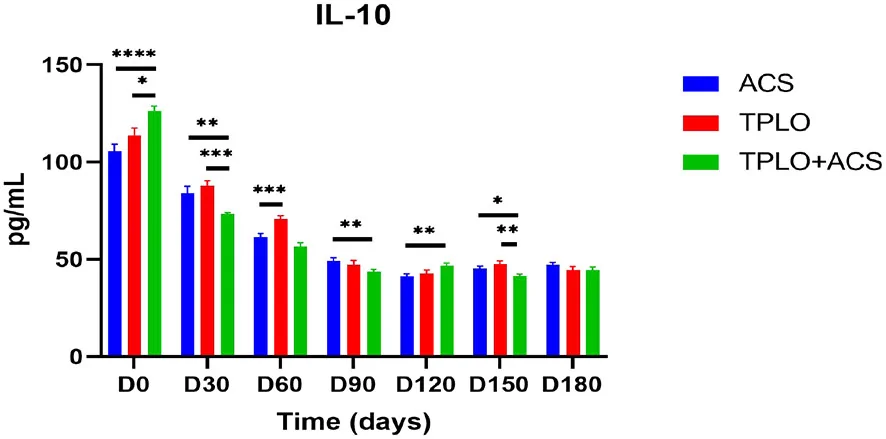
Changes in mean IL-10 concentrations in stifle joint synovial fluid. The results are presented as the ± SD, and statistical significance was calculated by *t* tests for two groups and one-way ANOVA for three groups. *p < 0.05, **p < 0.01, ***p < 0.001, ACS, autologous conditioned serum, TPLO, tibial plateau leveling osteotomy, *n* = 20 per group.

The IL-10 (anti-inflammatory cytokine) levels in all three groups exhibited a general declining trend over the 180-day postoperative period. At baseline (D0), the TPLO+ACS group exhibited significantly higher synovial IL-10 concentrations compared to both the ACS and TPLO groups (*p* < 0.001). The TPLO+ACS group maintained significantly elevated IL-10 levels relative to the other two groups at D30 and D60 (*p* < 0.01). From D90 to D120, the TPLO+ACS group continued to show significantly higher IL-10 concentrations compared to the TPLO group (*p* < 0.01), while no significant differences were observed between the TPLO+ACS and ACS groups during this period. At D150, the TPLO+ACS group still demonstrated significantly higher IL-10 levels than the TPLO group (*p* < 0.05). By D180, IL-10 concentrations in all three groups decreased to a similar range (45 pg/mL), with no statistically significant differences among them (Figure 6).

**Figure 7:**
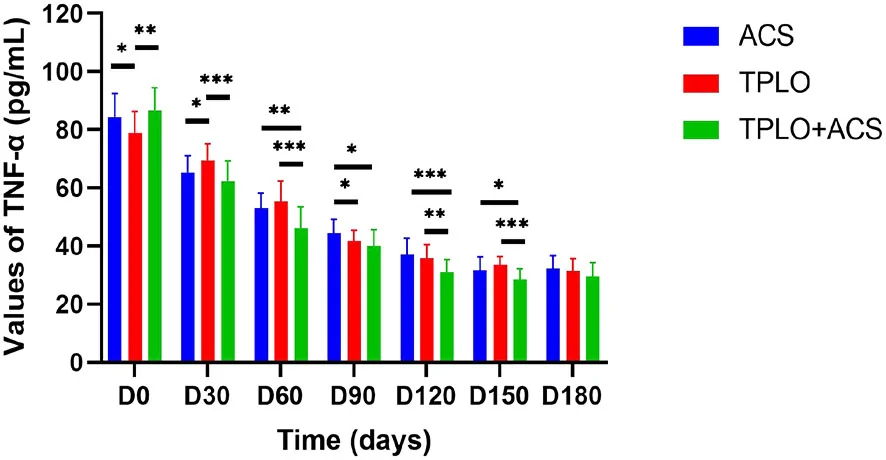
Changes in mean TNF-α concentrations in stifle joint synovial fluid. The results are presented as the mean ± SD, and statistical significance was calculated by *t* tests for two groups and one-way ANOVA for three groups. *p < 0.05, **p < 0.01, ***p < 0.001, ACS, autologous conditioned serum, TPLO, tibial plateau leveling osteotomy, *n* = 20 per group.

As another key pro-inflammatory cytokine, TNF-α levels in all three groups exhibited a general declining trend over the 180-day postoperative period. At baseline (D0), synovial TNF-α concentrations were comparable among the ACS, TPLO, and TPLO+ACS groups, with the TPLO+ACS group showing a slightly but significantly higher level than the TPLO group (*p* < 0.01). The TPLO+ACS group demonstrated significantly lower TNF-α values compared to the TPLO group at multiple time points (D30, D60, D90, D120, D150; *p* < 0.001, *p* < 0.01, *p* < 0.05, respectively). Additionally, the TPLO+ACS group showed significantly reduced TNF-α levels relative to the ACS group at D30 and D60 (*p* < 0.01). The ACS group also exhibited significantly lower TNF-α concentrations than the TPLO group during the early postoperative phase (D30 and D60, *p* < 0.05). By D180, TNF-α levels in all three groups had decreased to a similar range (30 pg/mL), with no statistically significant differences observed among the groups (Figure 7).

### 3.5 Complications

Postoperative complications are summarized in Table 6. Complications were observed exclusively in the surgical groups (TPLO and TPLO+ACS, *n* = 20 per group), while no complications were reported in the ACS group.

**Table 6:** Postoperative complications of 60 stifle joints with CCLR.

Group	Unilateral/Bilateral Stifles(*n*/*n*)	Postoperative (D180)					
		Hindlimb edema	Seroma	Wound infection	Implant failure	Fibula fracture	Fracture nonunion
ACS	14/6	0	0	0	0	0	0
TPLO	12/8	13	6	0	0	6	0
TPLO+ACS	10/10	11	9	0	0	9	0

In the TPLO group, hindlimb edema was the most frequent complication (13/20, 65%), followed by fibular fracture (7/20, 35%) and seroma formation (6/20, 30%). No cases of wound infection, implant failure, or nonunion were documented. Similarly, in the TPLO+ACS group, hindlimb edema occurred in 11/20 (55%) of cases, seroma in 9/20 (45%), and fibular fracture in 5/20 (25%). No instances of wound infection or implant failure were noted in this group.

## 4 Discussion

This study compared the application of ACS monotherapy, TPLO, and combined TPLO+ACS in dogs with CCLR, with treatment efficacy critically assessed across three domains: orthopedic assessment score, radiographic osteoarthritis scoring, and synovial inflammatory cytokine levels.

### 4.1 Functional recovery kinetics and the influence of body weight and complication

The three treatments demonstrated distinct recovery kinetics, as evidenced by dynamic alterations in OAS (including lameness, range of motion, and pain). Before day 90, the mean OAS scores for the TPLO and TPLO+ACS groups remained higher than those for the ACS group. The peak at day 30 in both osteotomy groups likely reflects the combination of surgical pain from the corrective osteotomy and the greater biomechanical stressexerted by heavier dogs on the stifle joint (7, 10). Conversely, after day 90, the mean OAS values shifted, with the TPLO+ACS group showing lower scores than the TPLO group, which in turn were lower than the ACS group. This pattern is similar to the findings of Shimada et al. (2020), who reported that peak vertical forces (PVF) were significantly higher at 6 months postoperatively than preoperatively across all groups (7). The TPLO+ACS group achieved the lowest final OAS at day 180, indicating superior functional recovery of osteoarticular function compared to monotherapy.

Notably, there were significant baseline differences in body weight among the three groups, with the ACS group exhibiting a lower mean body weight than the TPLO and TPLO+ACS groups. Body weight is a well-recognized confounder in canine CCLD research, as it affects both cruciate failure risk and the complication profile of surgical management (11, 12). This allocation bias was further reinforced by clinical practice patterns in small-breed dogs, where owner preference and lower body mass often steer cases toward conservative or injectable biologic therapy rather than osteotomy (13, 14). Consequently, body weight must be considered a potential confounding factor when interpreting outcomes, particularly regarding postoperative functional recovery and complications such as seroma and soft tissue swelling observed in this study. Because both the TPLO and TPLO+ACS groups underwent tibial osteotomy, there is an inherent probability of procedure-associated complications, including osteotomy-site morbidity and soft-tissue events (15). In contrast, the ACS group consisted of dogs with lower body weights that received intra-articular ACS injections only; therefore, this group did not experience the types of surgical complications tracked in our statistical analysis.

In this study, minor complications such as seroma formation and soft tissue swelling were treated by applying an ice pack for 15–20 min every 4 h. Intraoperative fibula fractures, which are a recognized complication of TPLO resulting from inadvertent saw blade contact during osteotomy, typically do not require specific intervention.

### 4.2 Long-term radiographic OA progression

No significant OA progression was detected within the first 180 days in any group, indicating minimal short-term radiographic changes regardless of treatment modality. However, long-term follow-up up to 720 days revealed a significant time–treatment interaction, underscoring divergent trajectories of OA progression among the three protocols. Notably, the TPLO+ACS group maintained significantly lower OA scores from 360 days onward compared to the ACS group, and by 720 days, its radiographic scores were also significantly lower than those of the TPLO group. Intragroup analyses further demonstrated that while both ACS and TPLO groups exhibited significant OA progression from baseline to 2 years—consistent with the progressive radiographic OA reported after standalone TPLO in uncomplicated CCLR cases (7)—the TPLO+ACS group showed no significant radiographic deterioration over the same period. These imaging findings indicate that adjunctive intra-articular ACS combined with TPLO mitigates long-term OA progression relative to either monotherapy, in line with prior evidence that autologous conditioned serum modulates synovial inflammation in canine OA (e.g., Soontararak et al., 2022) and that surgical stabilization alone does not arrest OA progression (5, 7).

### 4.3 Synovial cytokine modulation and its clinical impact

While all groups showed a general decline in synovial cytokines over 180 days, the TPLO+ACS group achieved significant reductions in key pro-inflammatory cytokines (IL-1β and TNF-α) and maintained a more favorable anti-inflammatory profile. Although autologous conditioned serum is principally characterized by enrichment of interleukin-1 receptor antagonist (IL-1Ra) rather than a consistent elevation of IL-10 (5), the TPLO+ACS group in the present study additionally exhibited sustained IL-10 levels that were significantly higher than those in the TPLO group from day 90 onward. Coupled with the suppression of IL-1β and TNF-α, this pattern indicates a balanced cytokine milieu. By mitigating the early postoperative inflammatory surge and sustaining protective anti-inflammatory signaling—while IL-1Ra from ACS neutralizes residual IL-1β activity—the combined protocol created a microenvironment conducive to chondroprotection and soft-tissue repair. In contrast, the TPLO group exhibited the least favorable inflammatory profile, with persistently unbalanced cytokine levels throughout the observation period. Although the ACS group demonstrated better early anti-inflammatory effects than the TPLO group (significantly lower IL-1β and TNF-α at days 30 and 60), its efficacy in maintaining elevated IL-10 was not statistically superior to TPLO+ACS beyond

day 90. This indicates that while a single biological treatment offers short-term benefits, the mechanical stabilization provided by TPLO appears to be a prerequisite for the biologic agent to sustain its modulatory effects on the synovial milieu, a principle consistent with the broader literature on adjuvant biologics following stifle stabilization (5).

### 4.4 Integrated mechanisms

The convergence of all cytokine levels to comparable ranges by day 180 aligns with the natural resolution of acute inflammation in post-traumatic OA. However, the TPLO+ACS group's ability to maintain a more balanced cytokine milieu during the D30–D150 period likely contributes to the better functional outcomes and delayed radiographic OA progression reported in our clinical assessments. The radiographic outcomes in the TPLO+ACS group reflect a greater effect: TPLO provides mechanical stability, while ACS modulates the synovial inflammatory microenvironment—as supported by *in vitro* evidence that ACS enriches IL-1Ra and suppresses IL-1β-driven inflammation (5)—and our data further show concurrent suppression of TNF-α with sustained IL-10 elevation in the early-to-mid postoperative phase. This multidimensional immunoregulatory mechanism created a more favorable milieu for chondroprotection and slowed OA progress. In contrast, the ACS group, despite showing early anti-inflammatory benefits attributable to ACS biologic properties (5), lacked mechanical stabilization and thus failed to prevent late OA progression. The TPLO group, although mechanically sound, did not benefit from the anti-inflammatory modulation of ACS, resulting in a steady increase in OA scores over time (7). These results highlight that neither mechanical nor biological intervention alone is sufficient to durably delay OA progression; rather, their combination is required to achieve optimal long-term joint preservation.

This study has several limitations, including a small sample size and significant intergroup differences in body weight. Due to resource constraints, non-traumatic postoperative knee examinations (such as 1.5T MRI) could not be performed to rule out confounding factors like meniscal injuries, Concomitant meniscal tears, which commonly occur secondary to CCLR in large dogs, significantly impair postoperative limb function and accelerate the progression of osteoarthritis. Future research should further explore the chondroprotective mechanisms of ACS.

## 5 Conclusions

This study establishes that the combination of TPLO with intra-articular ACS yields superior outcomes for canine CCLR compared to monotherapy: TPLO restores biomechanical homeostasis, while ACS effectively suppress the release of pro-inflammatory cytokines (IL-1β and TNF-α) while maintaining elevated levels of the anti-inflammatory cytokine IL-10, thereby accelerating functional recovery and mitigating OA progression. In summary, TPLO+ACS represents the most efficacious strategy for managing CCLR, providing a dual benefit of structural stabilization and biological optimization.

## Data Availability

The original contributions presented in the study are included in the article/supplementary material, further inquiries can be directed to the corresponding author.
